# Dispersion Energies with the i-DMFT Method

**DOI:** 10.1021/acs.jctc.4c00219

**Published:** 2024-06-25

**Authors:** Yihan Hu, Xiaowei Sheng, Jian Wang, Evert Jan Baerends

**Affiliations:** † Anhui Province Key Laboratory for Control and Applications of Optoelectronic Information Materials, Anhui Normal University, Anhui, Wuhu 241000, China; ‡ School of Science, Huzhou University, Zhejiang 313000, China; § Section Theoretical Chemistry, Faculty of Science, Vrije Universiteit, De Boelelaan 1108, Amsterdam 1081HV, The Netherlands

## Abstract

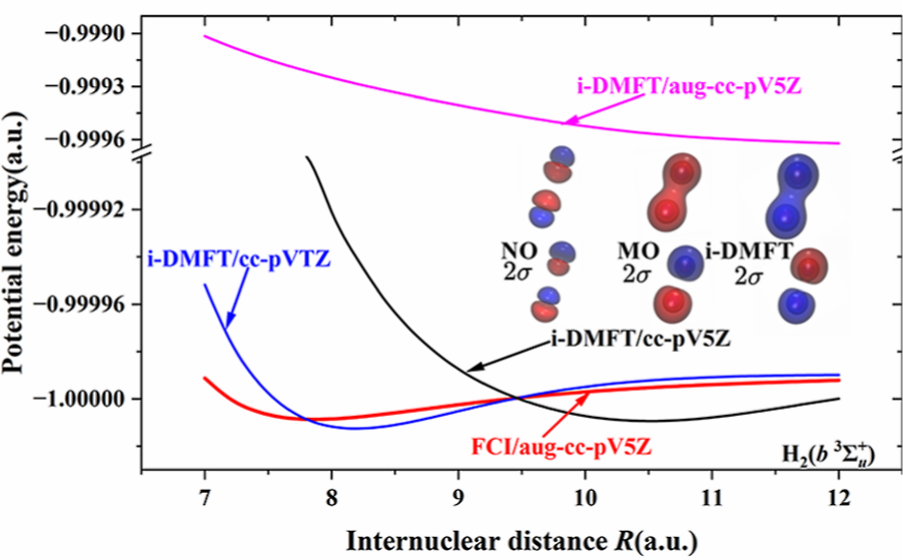

The recently proposed i-DMFT method [Wang and Baerends, *Phys. Rev. Lett.***128,** 013001 (2022)] has been proven to be ideally suited to recover strong static correlation in dissociating covalent bonds. Here, the application to van der Waals bonding is investigated using the prototype van der Waals systems triplet H_2_ and ground-state He_2_. It is demonstrated that the i-DMFT orbitals are in this case essentially different from the natural orbitals, and the i-DMFT occupations differ substantially from the NO occupations. This is shown to lead to rather deficient interaction potential curves, even if a reasonable well depth may be obtained by fitting of parameters. If the basis set is extended, however, it may no longer be possible to generate van der Waals bonding at all. The linear behavior of the two-electron cumulant energy *E*_cum_ as a function of the “entropy” *S* along a dissociation coordinate, which was the basis of i-DMFT, is distinctly poorer in the case of van der Waals bonding than for covalent bonding.

## 1 Introduction

The recently introduced i-DMFT method for the calculation of potential energy surfaces of molecules^1^ has proved to work very well for geometric distortions of covalently bound systems.^1−4^ In these systems, left–right correlation, which becomes at elongated bond lengths nondynamical correlation in nature, is the dominant correlation type. The question has been raised if also a dynamical correlation such as dispersion interaction in van der Waals molecules might be covered. A first attempt proved reasonably successful.^1^ In this paper, we consider this question in more detail and will arrive at the conclusion that dispersion interaction falls outside the scope of the i-DMFT method in its present formulation.

After a brief account of the i-DMFT method in Section 2, we address the issue of dispersion energies in Section 3. It is argued that a major impediment to the calculation of dispersion energies by the i-DMFT method arises from the essential difference in this case between natural orbitals (NOs) and the orbitals generated in the i-DMFT method. Although the i-DMFT method shares with the exact one-electron reduced density matrix that it involves fractional occupations of orbitals, this does not imply that these orbitals are NOs. The difference between i-DMFT orbitals and NOs has been discussed before, see ref (1) (in particular the Supporting Information, see also ref (5)). The difference of the i-DMFT orbitals and the NOs proves to be crucial in the case of dispersion energies. Moreover, because the approximation of the two-electron cumulant energy with a linear expression in the entropy *S* has been the basis of i-DMFT, we investigate in Section 4 whether this linear relation holds for the cumulant energy in the special case of the dispersion energy. The linear relation is shown to be relatively poor in this case, which also indicates that i-DMFT cannot be applied as is to dispersion energies. These findings are substantiated in Section 5, which presents potential energy curves (PECs) for triplet H_2_ and ground-state He_2_ calculated with the i-DMFT method with various basis sets. These highlight the troublesome nature of the application of i-DMFT to the van der Waals bonding. They also show that problems may arise when very large basis sets are used. The findings are summarized in Section 6. In this paper, all FCI calculations have been performed with the GAMESS(US) code^6^ and the two-electron integrals for, e.g., the *E*_cum_ calculations are obtained from ref (7). The graphic for the table of contents is generated by Multiwfn^8^ and VMD.^9,10^

## 2 i-DMFT Method

The total electronic energy of a molecule can be written in terms of the one-particle reduced density matrix(1-RDM) γ(1, 1′) and the pair density Γ(1, 2) as
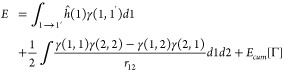
1where the two-electron cumulant energy *E*_cum_ is defined as the difference between the total electron–electron interaction energy and the Hartree–Fock (HF) like part depending on the 1-RDM
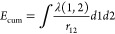
2with
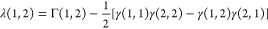
3*E*_cum_ can be called a correlation energy, but it is clearly different from the traditional correlation energy as used in quantum chemistry, which is the difference between exact total energy and Hartree–Fock energy
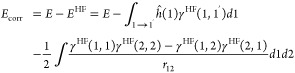
4The HF 1-DM γ^HF^ is different from the exact 1-RDM γ, in particular in cases of strong electron correlation.

It has been suggested by Collins^11^ that the traditional correlation energy would be related (be proportional) to an expression in terms of the natural orbital (NO) occupation numbers (ON) that was constructed in analogy to the information entropy as defined by Shannon^12^
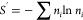
5where the occupation numbers are used instead of the probabilities *p*_*i*_ of events in Shannon’s theory of information. However, such a relation of *E*_corr_ with *S*′ proved to be nonexistent.^13,14^ But an investigation of *E*_cum_ at different internuclear distances in small molecules did prove it be linearly dependent on *S*′ with high accuracy.^15,16^ These results were obtained with close to exact wave functions (1-RDM and ONs, two-electron density matrix). It is of course desirable to exploit such a simple representation of the cumulant energy with an efficient self-consistent computational scheme to determine orbitals and occupation numbers. That cannot be based on *S*′ but one may exploit the fact^1,2^ that *E*_cum_ also exhibits a linear dependency along the dissociation coordinate *R* on the “entropy” *S* defined as
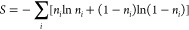
6which we write in terms of the parameters *K* and *D*
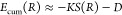
7*S* of (6) is in fact the true thermodynamic entropy of a gas of noninteracting Fermions occupying the available one-particle states with occupation numbers *n*_*i*_, dependent on the temperature of the gas. This is only a formal analogy because we are not dealing with a thermodynamic (macroscopic) system and there is no temperature.

We write the total energy as
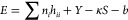
8where
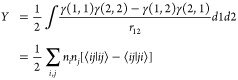
9We use the parameters κ and *b* to indicate that not necessarily the parameters *K* and *D* from the linear fit (7) will be used, but other means of obtaining these parameters will be considered, see below.

The total energy (8) can be optimized under variation of the orbitals and occupation numbers, under the constraints of orthonormality of the orbitals and total electron number *N*. This yields for the orbitals the one-electron equations
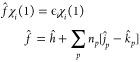
10and for the occupation numbers the Fermi–Dirac distribution
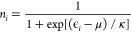
11(μ is determined by the condition that the number of electrons should be *N*).

It is clear that the one-electron Hamiltonian is very close to the Fock operator of Hartree–Fock theory. In particular, the above equations converge to those of the Hartree–Fock method when the occupation numbers approach 1.0 and 0.0. While the occupied Hartree–Fock orbitals are close to the strongly occupied NOs (*n*_*i*_ ≲ 1.0), this is not true in general for the virtual Hartree–Fock orbitals and the virtual NOs (*n*_*i*_ ≳ 0.0). (We will call the weakly occupied NOs with occupation numbers 0.5 > *n*_*i*_ > 0.0 just “virtual” orbitals.) The complications which arise from this circumstance are considered in Section 3.

## 3 i-DMFT Orbitals versus Natural Orbitals

In principle, the constants κ and *b* in eq 8 are functionals of the orbitals or the full one-electron density matrix, but they have not been obtained from first principles. Rather they have until now been determined from fitting the expression—*κS*(*R*) – *b* to the wave function quantity *E*_cum_ (*R*) or alternatively by solving from the total energy (either from theory or from experiment) at two geometries, such as −*D*_e_ at the equilibrium geometry and zero at dissociation. The subsequently calculated full potential energy curves (PECs) proved to be very accurate,^1,2^ i.e., very close to the PECs from wave function calculations (FCI) in the same basis set. In the dissociation cases studied, where large changes occur to the occupation numbers of the originally almost fully occupied and originally almost unoccupied orbitals, it was found that the i-DMFT occupation numbers were very close to the NO ONs.^1,2,4^ These are cases where the “active” NOs, the ones that change their occupation numbers significantly, are similar to the i-DMFT orbitals. “Similar” does not mean identical. As an example one may consider the prototype case of bond breaking in H_2_. In this case, one has a bonding combination of AOs and a corresponding antibonding combination, such as the σ_*g*_ and σ_*u*_ orbitals *N*_±_(1s_*A*_ ± 1s_*B*_). At long distance, these should be practically + and – combinations of the atomic 1s orbitals, and this is indeed the shape of the two corresponding NOs. The Fock operator, however, is such that quantitative differences arise. The occupied 1σ_*g*_ HF orbital is too diffuse, increasingly so when the bond distance increases. The too diffuse nature of the occupied HF orbitals and density in strong correlation cases such as stretched H_2_ but also in multiple bond cases like N_2_ is well known,^17,18^ and this deviation from the strongly occupied NOs has been discussed in ref (1) (Supporting Information). The virtual HF orbitals are typically even much more diffuse, representing an added electron.^19^ The orbital energies of the HF 1σ_*g*_ and 1σ_*u*_ do not tend at large *R* to the atomic −0.5 au but to ca. −0.2 au, indicating the too diffuse character (and wrong asymptotic behavior). The same behavior occurs for the i-DMFT orbitals. This can be remedied by replacing the constant κ with a functional of the orbitals and occupation numbers, see ref (5). Nevertheless, the fitting of κ to the desired properties (such as *D*_e_) apparently masks these differences of detail between the NOs and the i-DMFT orbitals, so i-DMFT is able to produce excellent potential energy curves (PECs).

This good behavior of i-DMFT can be understood when the active NOs are sufficiently similar to HF orbitals, as is the case for the left–right correlation situation exemplified by the dissociating H_2_. Similar good behavior can be expected in general when NOs and HF orbitals have the same shape. A point in case is the HeH^+^ molecule, where at dissociation the molecule (He with a proton at some distance) is well described in the HF model and, contrary to expectations,^20^ i-DMFT works well.^5^ The use of an entropic energy term was earlier employed in his TAO–DFT by Chai^21,22^ and in a recent work by Mazziotti and co-workers.^23−25^ In these works, where the density matrix functional for correlation is added to a DFT one-electron model rather than to Hartree–Fock, it is similarly the static correlation that is targeted.

However, there are also cases where the active NOs are essentially different from HF orbitals. This happens for dispersion interactions. An example is triplet H_2_ in its ^3^Σ_*u*_^+^ state: two up spin H atoms which exhibit dispersion interactions leading to a van der Waals minimum at 7.8 Bohr. This is a clear-cut case of dispersion interactions because there is no on-site dynamical correlation. In a case like He_2_, the on-site correlation of a single He atom is much larger than the dispersion energy, making it more complicated to distinguish the different effects. The ^3^H_2_ has both 1σ_*g*_α and 1σ_*u*_α fully (strongly) occupied. It has been shown^26^ that an accurate description of the dispersion energy requires only very few NOs. Only the double excitation to the |2σ_*g*_α2σ_*u*_α| ^3^Σ_*u*_^+^ state and the ^3^Σ_*u*_^+^ state belonging to the doubly excited  configuration contribute significantly to the dispersion energy. Analyzing the wave function in terms of the two-electron density matrix, in particular the pair density, shows that the dispersion can be described as a dynamical polarization of one H atom, H_*a*_ say, due to the presence at some position of the electron at the H_*b*_ atom. When the electron at H_*b*_ is at the internuclear axis there is a small dipolar distortion of the spherical electron density at H_*a*_ in the direction of the axis, as seen in Figure 2 of ref (26). (For off-axis positions of the reference electron, polarization perpendicular to the axis comes into play.) As observed in ref (26), it is striking that the 2σ_*g*_ and 2σ_*u*_ NOs consist predominantly of H p_σ_ like orbitals (directed along the bond), in contrast to the HF (and KS) 2σ_*g*_ and 2σ_*u*_ orbitals, which have 2s nature. The p_σ_ character of the 2σ_*g*_ and 2σ_*u*_ orbitals is not just H 2p like, but actually it involves more contracted basis functions,^26^ in accordance with the fact that they describe dispersion-type polarization of the 1s density of the H atom.

**Figure 1:**
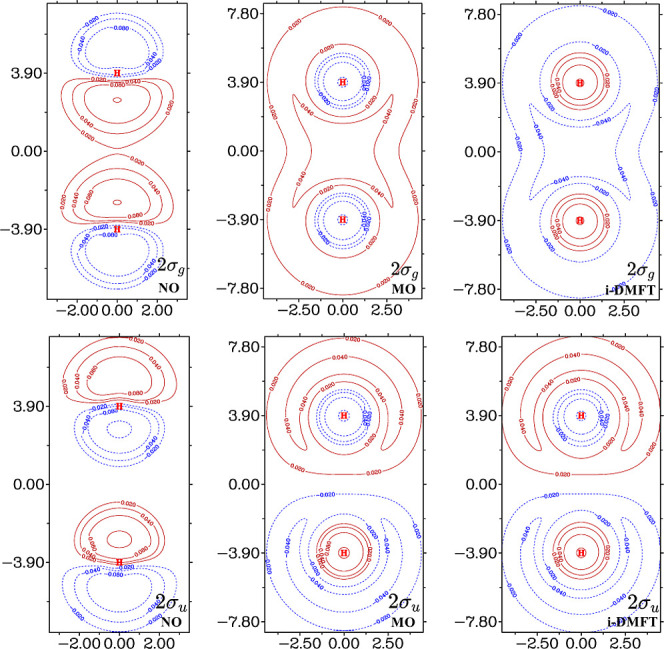
Contour plots of the 2σ_*g*_ (upper panels) and the 2σ_*u*_ (lower panels) orbitals of ^3^H_2_ from cc-pVTZ basis set. NOs from FCI: left column; HF MOs: middle column; i-DMFT orbitals: right column. The plots were generated with Multiwfn,^8^ distances in Bohr.

These points are illustrated in Figure 1, which displays in the first column the 2σ_*g*_ (upper panel) and 2σ_*u*_ (lower panel) NOs of ^3^H_2_ from a full configuration interaction (FCI) calculation in a cc-pVTZ Gaussian basis set. Compared to the 2σ_*g*,*u*_ Hartree–Fock MOs (second column), it is clear that these orbitals are very different in character. Whereas the NOs clearly have p_σ_ characters, the HF MOs are 2s-like combinations (note the tail close to the nucleus for orthogonality on the 1s which has a different sign than the extensive 2s valence region). The i-DMFT orbitals (third column) look very much like the HF MOs, i.e., they are very different from the NOs. This implies that the i-DMFT orbitals are not suitable to describe the dispersion-type electron correlation.

In Table 1, some numerical characteristics of the orbitals are given. We note that the HF orbital energies for the 1σ_*g*_ and 1σ_*u*_ are very close to −0.5, as is to be expected for a system that consists almost of two single H atoms with only small perturbations due to Pauli repulsion between the two up-spin atoms and a small attractive dispersion energy. This is true for all basis sets, indicating that the cc-pVTZ is already an adequate basis set for the description of the occupied 1σ orbitals. However, the 2σ_*g*_ and 2σ_*u*_ HF orbital energies are positive in the cc-pVTZ basis, and become considerably lower but remain positive in the cc-pVT5Z basis, with again a large lowering (approaching 0.0) in the aug-cc-pV5Z basis. This is a well-known phenomenon for virtual HF orbitals of small molecules, indicating their unrealistic nature: with positive orbital energies they are in principle infinitely extended orbitals for unbound electrons, plane wave like with some orthogonality wiggles over the molecular domain. So in a finite basis they will lower their energy toward zero given sufficient flexibility in the basis set, as seen in ref (19). The i-DMFT orbital energies are similar to the HF ones, as can be expected from the occupation numbers of the i-DMFT orbitals being almost 1.0 for the 1σ orbitals and very small for the 2σ orbitals. Therefore, the “Fock” operator for the i-DMFT orbitals is very close to the HF Fock operator in this case. Turning to the NOs from the FCI calculations, it is clear that the dispersion interaction is reflected in only small deviations of the NO occupation numbers from 1.0 for 1σ orbitals and 0.0 for 2σ orbitals (but these 2σ ONs are crucial for the dispersion energy, as seen in ref (26)). It should be kept in mind, however, that, as we have seen, the 2σ NOs are very different orbitals than the 2σ HF and i-DMFT orbitals. Comparing nevertheless the occupations of the i-DMFT orbitals with the NO ONs, we note large differences: the i-DMFT model with its Fermi–Dirac distribution shifts much more population to the 2σ orbitals, the more so when the orbital energies of those orbitals are lowered in the larger basis sets. The effect is particularly striking in the aug-cc-pV5Z basis where the energy lowering of the virtual orbitals combined with the Fermi–Dirac occupation scheme leads to relatively large occupation of the virtual (note also the striking lowering of the 1σ_*g*,*u*_ populations).

**Table 1:** Orbital Energies in au (for Hartree–Fock and i-DMFT) and Occupation Numbers (for NOs and i-DMFT Orbitals) for ^3^H_2_ at the van der Waals Distance *R* = 7.8 Bohr, for the Basis Sets cc-pVTZ, cc-pV5Z and aug-cc-pV5Z

^3^H_2_	1σ_*g*_	1σ_*u*_	2σ_*g*_	2σ_*u*_
cc-pVTZ				
HF orb. en.	–0.502	–0.498	0.322	0.380
i-DMFT orb. en.	–0.500	–0.496	0.292	0.352
FCI occ. no.	0.999993	0.999993	0.459 × 10^–5^	0.459 × 10^–5^
i-DMFT occ. no.	0.994286	0.993976	786.5 × 10^–5^	370.3 × 10^–5^
cc-pV5Z				
HF orb. en.	–0.502	–0.498	0.164	0.203
i-DMFT orb. en.	–0.498	–0.494	0.142	0.176
FCI occ. no.	0.999980	0.999980	1.389 × 10^–5^	1.389 × 10^–5^
i-DMFT occ. no.	0.983708	0.982814	1839.54 × 10^–5^	1208.49 × 10^–5^
aug-cc-pV5Z				
HF orb. en	–0.502	–0.498	0.042	0.051
i-DMFT orb.en	–0.500	–0.495	0.027	0.037
FCI occ. no.	0.999963	0.999963	2.356 × 10^–5^	2.356 × 10^–5^
i-DMFT occ. no.	0.938041	0.934478	1934.85 × 10^–5^	1702.17 × 10^–5^

So from the orbital shapes, orbital energies, and occupation numbers of the i-DMFT model, we infer that the dispersion energy is a type of correlation energy that is not adequately treated with i-DMFT. We may add that there are other cases where the HF type of virtual orbitals differ from the “virtual” NOs. A point in case is the He atom, where the HF 2s orbital is very diffuse (Rydberg like) while the 2s NO is much more contracted. The 2s NO describes the dynamic correlation of the 1s electrons in He. It has accordingly the same spatial extent as the 1s orbital. Davidson pointed out in ref (27), p78, this difference between the HF and NO 2s orbitals: the 2s NO has a node at 0.9 Bohr and maximum at 1.8 Bohr, while the HF 2s orbital has a node at 1.5 Bohr and maximum at 4.4 Bohr. The 2s NO is suitable for describing the dynamical correlation among the 1s electrons in the He atom,^28^ but the HF 2s is much too diffuse for that purpose.

## 4 Two-Electron Cumulant Energy *E*_cum_ and the “Entropy” *S* for the Dispersion Energy in ^3^H_2_ and He_2_

**Figure 2:**
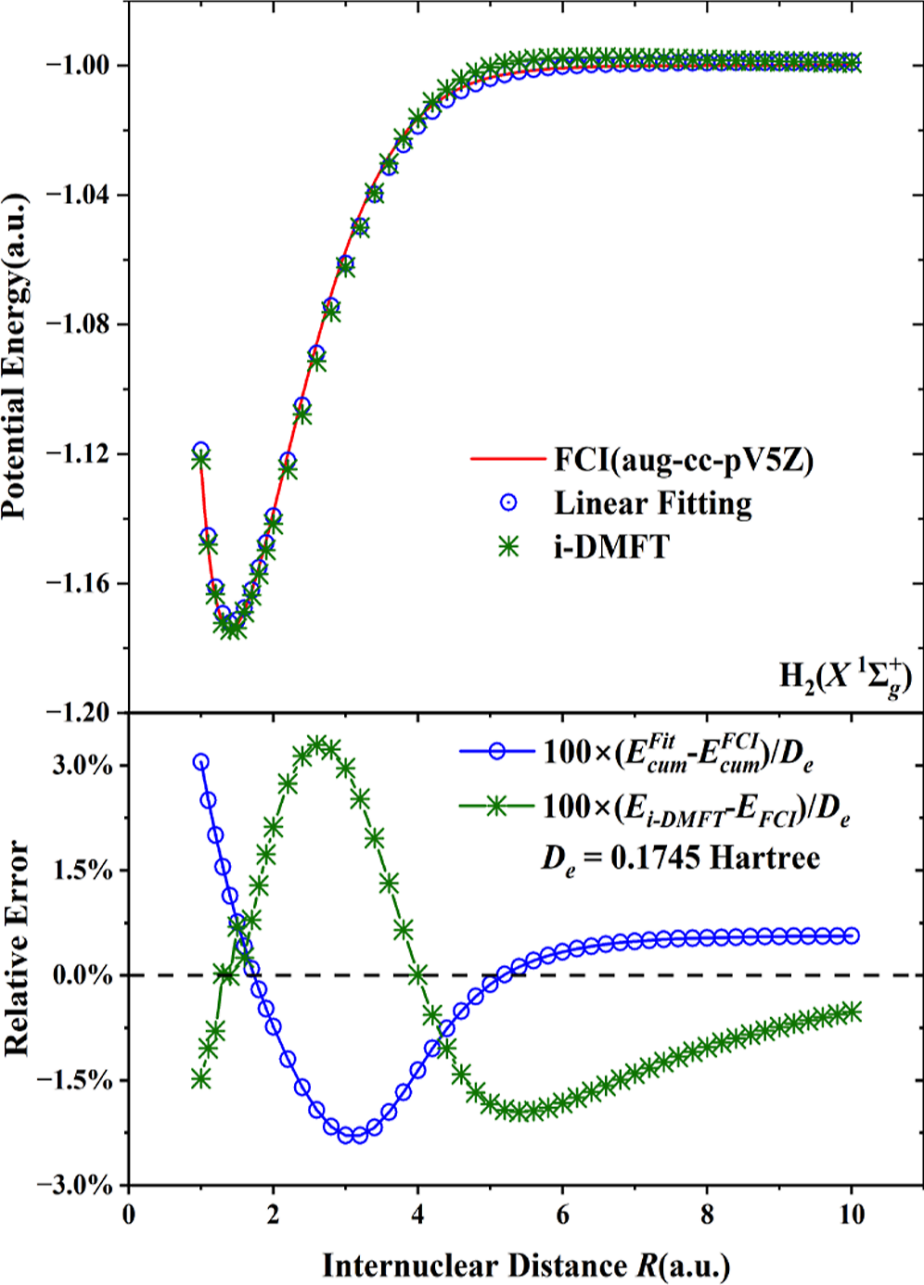
Upper panel: comparisons of the potential energy curve of the singlet ground state of H_2_ from FCI calculations with an aug-cc-pV5Z basis set (red curve) on the one hand and the linear fitting of *E*_cum_ (blue circles) and i-DMFT calculations (green crosses) on the other hand. Linear fitting means that the *E*_cum_ term in the total energy is replaced with the linear approximation (7), where the fitting parameters *K* and *D* are 0.0940 and 0.0508, respectively. Lower panel: relative errors with respect to FCI (as a percentage of the well depth *D*_e_) of the interaction potential of the i-DMFT calculation (green crosses) and of the linear fit to *E*_cum_ (blue circles). i-DMFT parameters for the aug-cc-pV5Z basis: κ = 0.08220, *b* = −0.02177137.

Given our finding there is a fundamental problem with the calculation of dispersion energies with the i-DMFT method, it is interesting to investigate what exactly is the relation between *E*_cum_ and the entropy-like function *S* (6) of the occupation numbers in this case. The i-DMFT method makes essential use of the linear relation between the cumulant energy *E*_cum_ and *S* of eq 6. This holds for cases of covalent bonding, as is illustrated for ground-state singlet H_2_ in Figure 2. The energy curve for singlet H_2_ computed with FCI (red curve) is compared to calculations where only the FCI *E*_cum_ term is replaced with a linear fit to *S* of (6) (blue circles). Moreover, the self-consistent i-DMFT results are shown (green crosses) with the parameters κ and *b* obtained by fitting the energy at *R*_e_ to *D*_e_, yielding κ = 0.08220 for the aug-cc-pV5Z basis.

**Figure 3:**
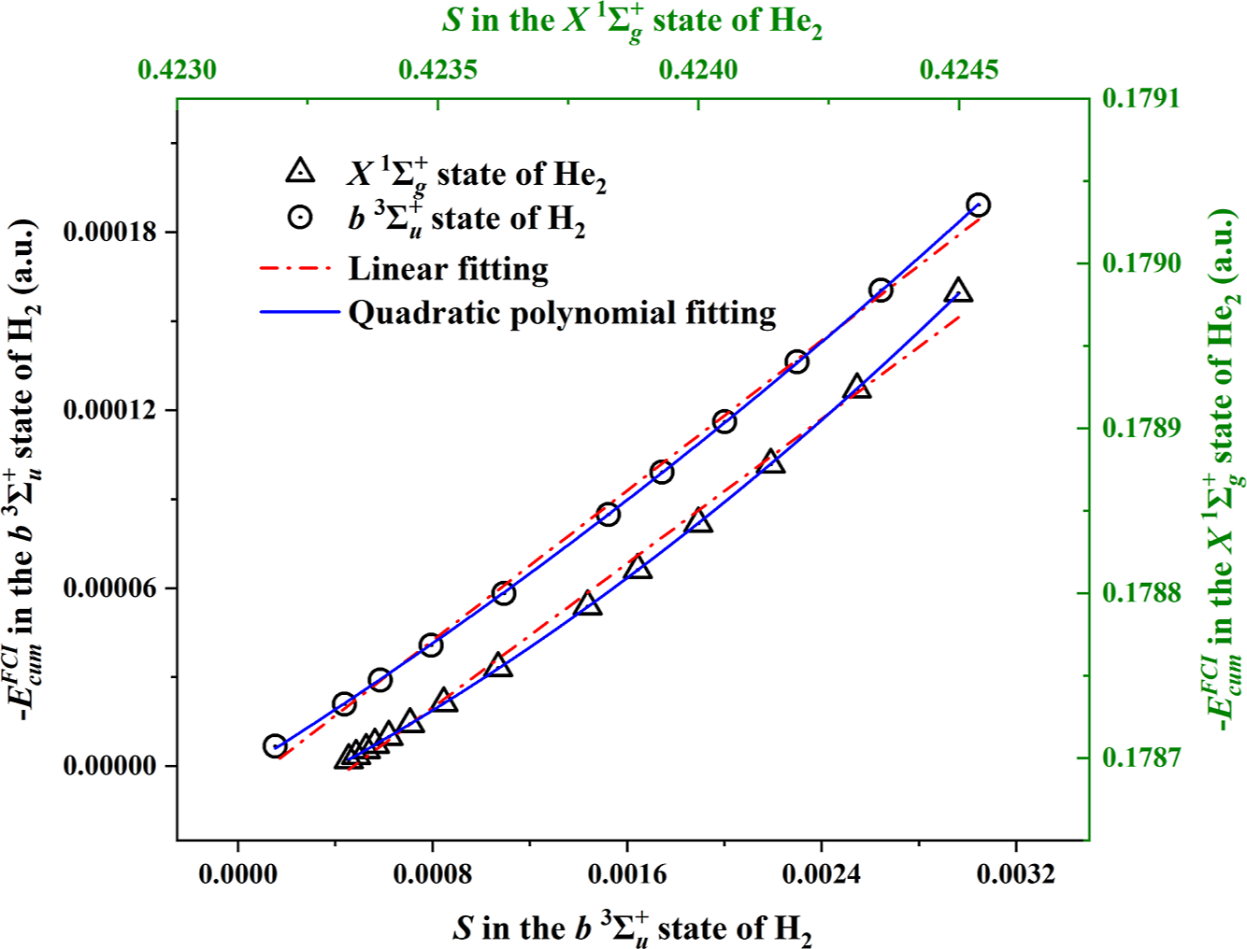
Two-electron cumulant energy *E*_cum_ from full CI in the aug-cc-pV5Z basis in the lowest triplet state of H_2_ (blue circles) and for the ground state of He_2_ as a function of the entropy *S* as defined in eq 6. The red dash-dotted line is the linear fit, the blue solid line is the quadratic fit. Bottom horizontal axis for *S* of triplet H_2_ and top horizontal axis for *S* of He_2_.

In order to judge the performance of i-DMFT, the relative error (as a percentage of the well depth *D*_e_ of the interaction potential) is given in the lower panel of Figure 2 (green crosses). The relative errors are less than 1.0% close to the equilibrium distance of 1.4 au, and ca. 3.0% maximum for a somewhat longer distance, trailing off to less than 1.0% again in the long distance limit. The linear fit has comparable errors, but of opposite sign. This good performance, and the close proximity of the FD distribution of i-DMFT occupation numbers to the NO ONs,^1,4^ rely on the linear relation of *E*_cum_ to *S*. However, the validity of this linear approximation for the van der Waals molecules is still an open question. The interaction energies in molecules like ^3^H_2_ and He_2_ are only in the order 10^–5^ au, much smaller than the errors of the linear fit in the covalent bonding case. So, we investigate if such a linear relation is also obtained in the dispersion energy cases of ^3^H_2_ and He_2_, as seen in Figure 3. It is clear from the figure that the linear fit is reasonably good, even though not perfect. It is to be noted that the dispersion energies are quite small, so the deviation of the linear fit is significant. This is numerically displayed in Tables 2 and 3, where the linear fit yields errors of ca. 15% of *D*_e_ for ^3^H_2_ around *R*_e_ = 7.8 au and ca. 25% for He_2_ around *R*_e_ = 5.6 au. Note that while *E*_cum_ is for ^3^H_2_ in the order of magnitude of the van der Waals well depth of 19.8 μH, it is in He_2_ very much larger than the VdW well depth of 34.8 μH because the on-site correlation between two electrons in one He atom, being ca. 41500 μH, is very much larger than the dispersion energy between two He atoms.

**Table 2:** Relative Error of the Cumulant Energy *E*_cum_ Calculated from Various Methods for the Lowest Excited Triplet State of H_2_^a^

*R*	*E*_cum_^FCI^	quadratic polynomial fitting (%)	linear fitting (%)
7.0	–0.0001890472	–2.162	24.481
7.2	–0.0001603366	0.786	7.336
7.4	–0.0001362559	1.735	–3.992
7.6	–0.0001160525	1.590	–10.964
7.8	–0.0000990866	0.928	–14.721
8.0	–0.0000848238	0.096	–16.150
8.5	–0.0000582186	–1.603	–13.547
9.0	–0.0000406854	–2.193	–6.904
9.5	–0.0000289460	–1.840	0.601
10.0	–0.0000209512	–0.932	7.621
12.0	–0.0000066812	3.595	26.239

a Here, the relative error is calculated by the formula 100 × (*E*_cum_^Fit^ – *E*_cum_^FCI^)/*D*_e_. *D*_e_ is the well depth of the interaction potential of H_2_, which is 19.8 μHatree. The FCI calculations are performed with aug-cc-pV5Z basis set applied.

**Table 3:** Relative Error of the Cumulant Energy *E*_cum_ Calculated from Various Methods for the Ground State of He_2_^a^

*R*	*E*_cum_^FCI^	quadratic polynomial fitting (%)	linear fitting (%)
5.0	–0.1789819696	1.292	42.503
5.2	–0.1789235773	–1.143	5.932
5.4	–0.1788779582	1.242	–13.689
5.6	–0.1788422145	–0.526	–22.652
5.8	–0.1788141086	0.277	–25.211
6.0	–0.1787919391	0.846	–24.183
6.5	–0.1787545576	1.043	–15.843
7.0	–0.1787332000	0.524	–6.816
7.5	–0.1787205978	0.214	0.518
8.0	–0.1787128840	0.118	5.955
8.5	–0.1787080092	0.042	9.726
9.0	–0.1787048451	–0.105	12.201
10.0	–0.1787013072	–0.482	14.835
12.0	–0.1786987185	–0.858	16.725

a Here, the relative error is calculated by the formula 100 × (*E*_cum_^Fit^ – *E*_cum_^FCI^)/*D*_e_. *D*_e_ is the well depth of the interaction potential of He_2_, which is 34.8369 μHatree. The FCI calculations are performed with aug-cc-pV5Z basis set applied.

**Figure 4:**
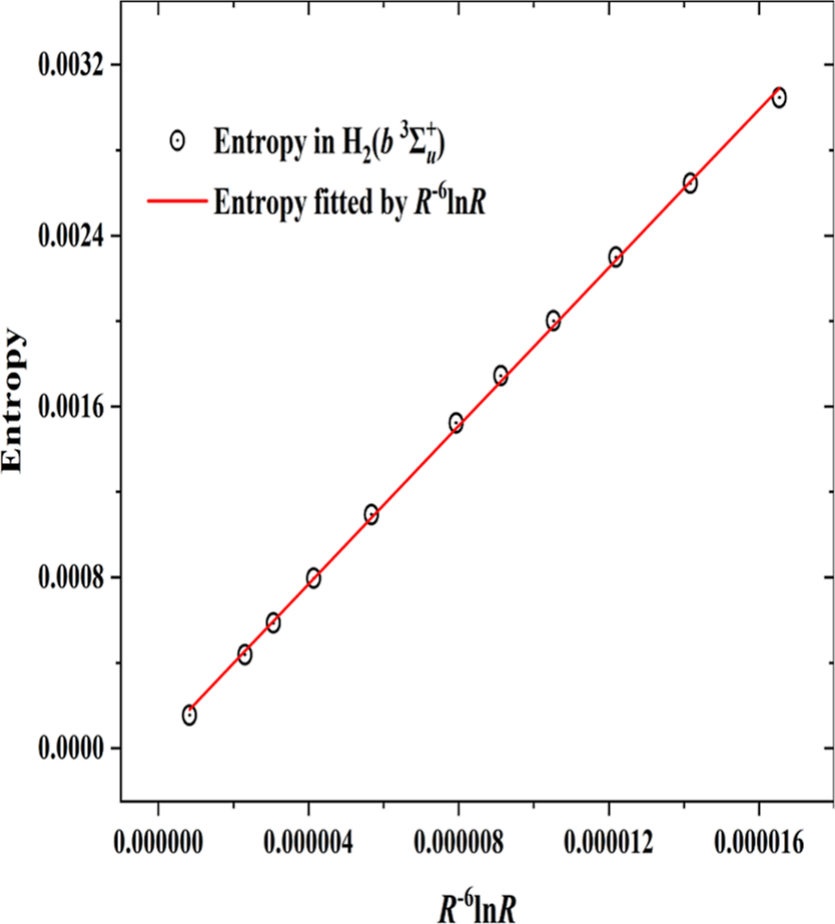
Internuclear distance *R* dependency of the entropy *S* in the *b*^3^Σ_*u*_^+^ state of H_2_. *S* is proportional to *R*^–6^ ln *R* starting from *R* = 7.0 Bohr (*R*^–6^ ln *R* = 0.00001654) to large *R*.

While the linear fit is deficient, one might consider a quadratic fit
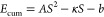
12Figure 3 suggests that a quadratic fit would be adequate, and Tables 2 and 3 demonstrate that the quadratic fit is accurate to approximately the 1% level. There is no numerical or analytic evidence for either linear or quadratic behavior of *E*_cum_ for VdW molecules. It has recently been deduced by Cioslowski et al.^29^ that *S* in H_2_ should go in the limit of very large *R* as *R*^–6^ ln *R*. This asymptotic behavior might not have been reached already around *R*_e_, but we have verified (see Figure 4) that indeed *S* is proportional to *R*^–6^ ln *R* over the whole range of the VdW well.

**Figure 5:**
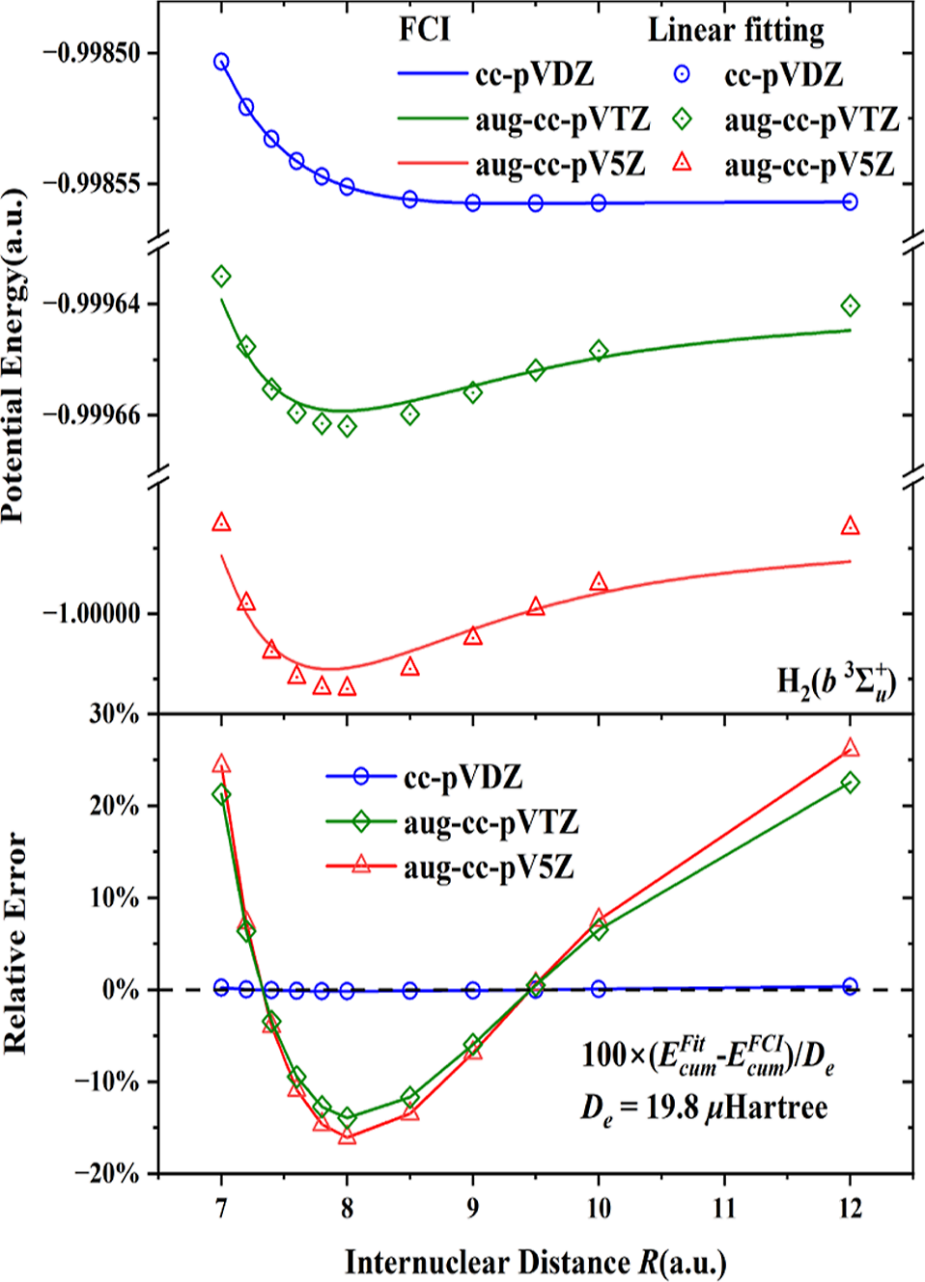
Upper panel: comparison of the potential energy curves of the *b*^3^Σ_*u*_^+^ state of H_2_ from the FCI and with the linear fitting of the *E*_cum_ term, with various basis sets. Lower panel: relative errors of the various basis sets. The values for the fitting parameters are listed in Table 4.

**Figure 6:**
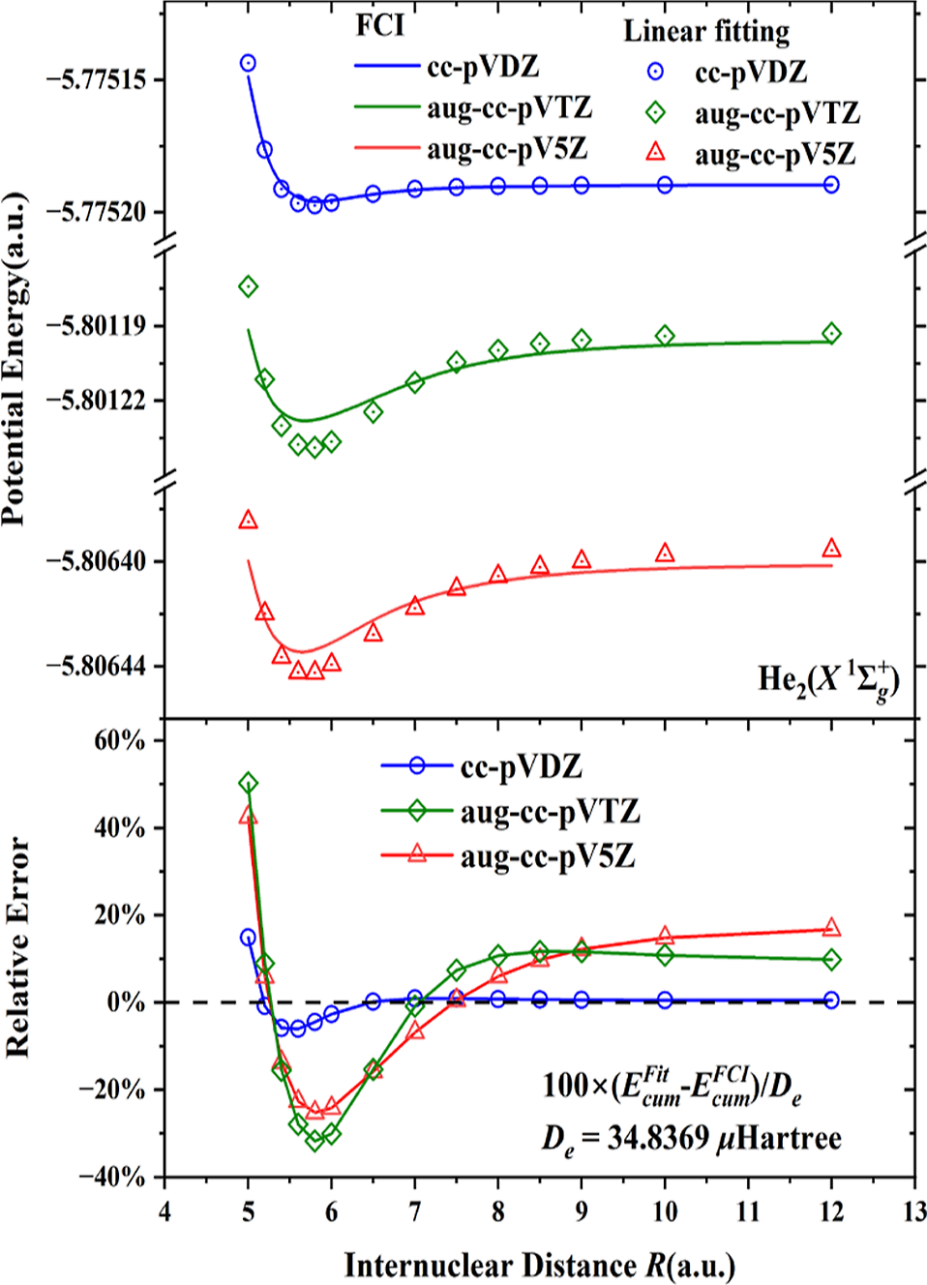
Upper panel: comparisons of the potential energy curves of *X*^1^Σ_*g*_^+^ state of He_2_ from the FCI and with the linear fitting of the *E*_cum_ term, with various basis sets. Lower panel: relative errors of the various basis sets. The values for the fitting parameters are listed in the Table 4.

The results so far have been obtained with the aug-cc-pV5Z basis. Figure 5 and 6 demonstrate the effect of the errors in the linear fit for potential energy curves for the *b*^3^Σ_*u*_^+^ state of H_2_ and *X*^1^Σ_*g*_^+^ state of He_2_, respectively. Here, all energy components are obtained from FCI calculations except for *E*_cum_, so these curves show the effect on the interaction potential of approximating the *E*_cum_ term of (1) with the linear fit. The basis set effect can be garnered from the curves for the smaller basis sets cc-pVDZ and aug-cc-pVTZ. For the very small basis set cc-pVDZ (in blue color), the linear equation of the entropy *S* is still a good approximation to the cumulant energy, but with such a small basis set the dispersion energy cannot be calculated reliably and a VdW minimum is hardly present. With increasing basis sets the FCI curves (green for aug-cc-pVTZ, red for aug-ccpV5Z) exhibit a clear VdW minimum, but the linear fitting of *E*_cum_ visibly deviates from the FCI calculations. In the lower panels of Figures 5 and 6, the relative errors occurring with the linear fitting are given. The discrepancy between the linear fitting and the cumulant energy are largest and negative (lower energy with the linear fit) around the equilibrium distances. For the *b*^3^Σ_*u*_^+^ state of H_2_ and the *X*^1^Σ_*g*_^+^ state of He_2_ the relative errors (with respect to *D*_e_) are over 15 and 20% respectively, in agreement with Tables 2 and 3. In addition, the errors of the linear fits do not disappear at long distances, the linear fit has a somewhat different long-range behavior compared to the FCI. It is clear that the dispersion interaction in the two van der Waals molecules is captured more adequately in the larger basis sets, aug-cc-pVTZ and aug-cc-pV5Z. The good agreement of FCI with the linear fit observed with the cc-pVDZ basis set is obtained just because of the very small percentages of dispersion interaction in the cumulant energy with that small basis. It is clear from Tables 2 and 3 that if the quadratic fitting would be used for the *E*_cum_ term, the behavior of the approximated curves would be much improved for the aug-cc-pVTZ and aug-cc-pV5Z bases (Table 4).

**Table 4:** Value of the linear Fitting Parameters to *E*_cum_ for *b*^3^Σ_*u*_^+^ State of H_2_ and *X*^1^Σ_*g*_^+^ State of He_2_ with Different Basis Sets Applied

basis set	*K*	*D*
H_2_		
cc-pVDZ	0.1093325246	–0.0000001344
aug-cc-pVTZ	0.0616915150	–0.0000071715
aug-cc-pV5Z	0.0631803069	–0.0000082079
He_2_		
cc-pVDZ	0.5354670689	–0.0561588275
aug-cc-pVTZ	0.2455569303	0.0680939799
aug-cc-pV5Z	0.2344498794	0.0794433648

The quadratic formula 12 leads to the same eigenvalue equations for the orbitals as eq 8, but a different equation for the Fermi–Dirac distribution
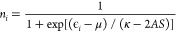
13The term 2*AS* introduces a modification to κ, which itself is different in the quadratic fitting. However, our tests so far indicate that this does not change the AO character of the i-DMFT 2σ_*g*,*u*_ orbitals to that of the NOs. The quadratic fitting of *E*_cum_ will, therefore, not lead to an improved description of the physics of the dispersion interaction in self-consistent i-DMFT calculations.

## 5 Application of I-DMFT for van der Waals Interaction

Although we have argued that i-DMFT is not suitable for van der Waals molecules, we can nevertheless investigate its performance for this purpose. The first test^1^ has been encouraging. Because the κ parameter is typically determined so that, e.g., the dissociation energy is obtained correctly, possible deficiencies may not be immediately apparent.

**Figure 7:**
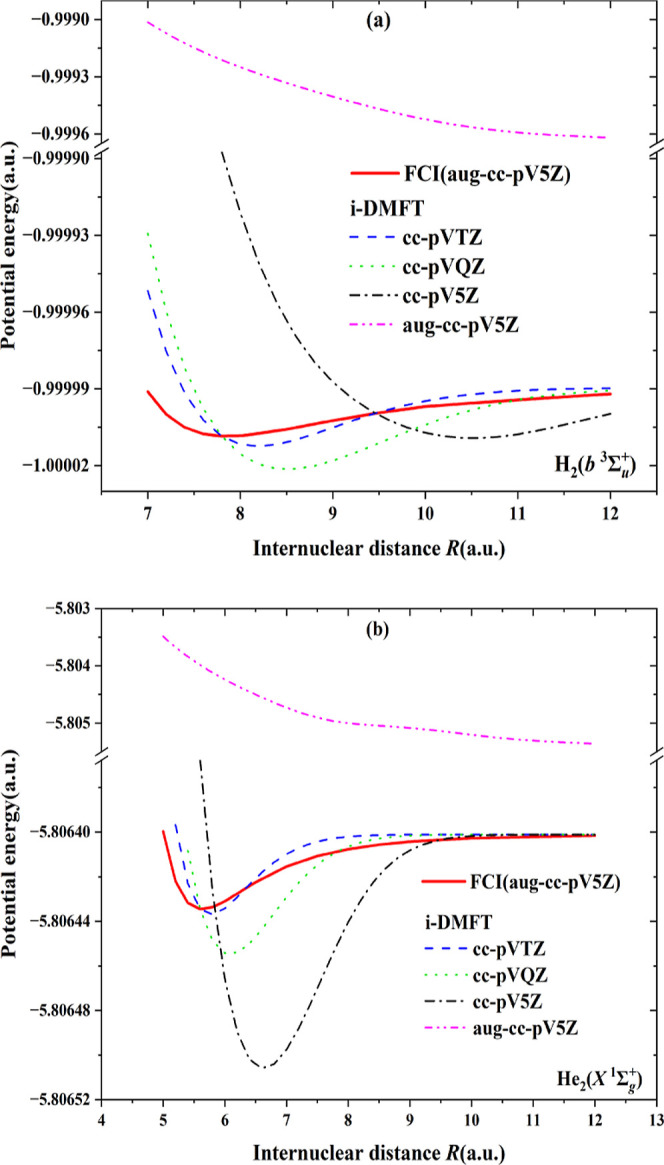
Potential energy curves (PECs) for triplet H_2_ (a) and He_2_ (b). The red drawn curve is the benchmark curve from FCI calculations in the largest basis (aug-cc-pV5Z). The blue, green, black, and magenta curves come from i-DMFT calculations with the basis sets cc-pVTZ, cc-PVQZ, cc-pV5Z, and aug-cc-pV5Z. See the text for choice of κ’s.

In Figure 7, it is demonstrated that it is possible to obtain potential energy curves (PECs) if the basis set is not too large. This is in spite of the fact that we have shown that the MOs in the i-DMFT differ from the NOs in the case of van der Waals bonding and, moreover, that the occupations of the i-DMFT MOs are very different from the NO ONs. The approximately correct depth of the van der Waals minimum is obtained in some cases (notably the smaller basis sets, cf. the cc-pVTZ basis in the figure) when using the standard choice of κ such that the energy at *R*_e_ is exactly equal to the FCI *D*_e_. The κ parameters in these calculations are listed in Table 5. However, with larger basis sets, it is no longer possible to determine κ such that a reasonable curve with a well depth of −*D*_e_ at *R*_e_ is obtained. At the chosen κ (close to the smaller basis set κ′s), the curves are distinctly different from the FCI curves: they are displaced, with (much) too deep minima (for cc-pV5Z basis) or entirely lacking a minimum at a reasonable bond length (the aug-cc-pV5Z basis). This situation is different from the studied cases of dissociation energies for covalently bound systems^1,4^ as well as the ionic system HeH^+^,^5^ which exhibit, in standard basis sets, i-DMFT MOs that are similar to the NOs and have accurate PECs and occupation numbers. We note from Table 1 that the occupation of the virtual orbitals (i.e., electron loss of the “occupied” orbitals) for the aug-cc-pV5Z basis has increased to 0.1275 el., which is much too high compared to the 7.4 × 10^–5^ for the NOs. This overoccupation of the virtual is a consequence of the appearance of an increasingly dense set of orbitals with energies around zero as solutions of the Fock operator when the basis set is very much expanded, particularly with diffuse functions.^19,30^ The Fermi–Dirac occupation scheme then forces (similar) occupations of all these orbitals. This leads—together with the wrong shape of the i-DMFT MOs—to too high population of these orbitals and too much increase of the energy. The further the basis set would be expanded, and the more orbitals with energies close to zero would appear, the more serious this problem of overpopulation of these states due to the Fermi–Dirac distribution would become. This is a caveat against an extreme basis set extension in the i-DMFT method.

**Table 5:** Value of the κ and *b* Parameters for the Curves in Figure 7

	H_2_		He_2_	
basis set	κ	*b*	κ	*b*
cc-pVTZ	0.07861	–0.0013560563	0.11631	0.0826665844
cc-pVQZ	0.07509	–0.0022401979	0.11242	0.081158705
cc-pV5Z	0.08	–0.0055761964	0.13522	0.0723116392
aug-cc-pV5Z	0.08	–0.0216793824	0.13522	0.0461026447

## 6 Summary

The possibility that the i-DMFT method might not only describe covalent bonds and their dissociation accurately but might also be able to provide reliable dispersion energies and thus be suitable for van der Waals bonding has been investigated in this paper. The conclusion is that i-DMFT fails in the latter case. This has been attributed to the fact that the “virtual” NOs are in this case truly different from the “virtual” i-DMFT MOs. While the latter are close to HF orbitals (in particular in this case where the occupation numbers differ very little from the 1.0 and 0.0 of HF), the NOs in the case of van der Waals bonding are qualitatively different. This has been highlighted for the case of triplet H_2_ where we find all i-DMFT MOs as expected to be close to HF MOs, whereas notably the 2σ_*g*_ and 2σ_*u*_ NOs are very different from the corresponding MOs, as seen in Figure 1. The NOs do not have the (2s ± 2s) character of the corresponding HF MOs but have (p_σ_ ± p_σ_) characters, where the p_σ_ character is much more contracted than the HF atomic 2p shape. This more contracted character is necessary in order to describe the polarization of the 1s charge density, which is the hallmark of the dispersion effect.^26^ Similarly, the π_*g*,*u*_ NOs (not shown) do not have atomic 2p_π_ ± 2p_π_ shapes but are more contracted. We also have pointed out that large basis sets exacerbate the problem because they lead to too large of an occupation of the “virtual” orbitals: the more of such orbitals are created in the virtual space due to an increase of the basis set, the higher the population of these orbitals will become due to the Fermi–Dirac occupation scheme of the i-DMFT method.

Cioslowski and Strasburger^31^ have very recently investigated if a linear Ansatz for *E*_cum_ such as in (8) could represent an exact 1RDM functional theory. They arrive—in a different way—at their eq 23, which is basically our eq 12 in ref (1), and conclude that this will not afford the exact 1RDM as solution. With the further derivation of the eigenvalue eq 10 and the Fermi–Dirac distribution (11), we have been able to explicitly demonstrate that indeed in the dispersion case the orbitals and occupation numbers of i-DMFT do not represent the exact 1RDM. The question whether the i-DMFT method is an exact 1RDM method has also been raised before, see ref (20). The present work underlines our earlier comment^5^ on this point: the solutions to the generalized Fock equation are not the NOs. For one thing, its “virtual” orbitals are not similarly contracted as often the first “virtual” (weakly occupied) NOs are, these NOs being effective for describing correlation of valence electrons. Our present results provide a specific example. They underline that the power of the i-DMFT method lies primarily in accurate rendering of the strong correlation effects (left–right correlation effects) arising from bond lengthening and dissociation when the AO character of the i-DMFT orbitals is similar to that of the NOs, as documented in the first applications.^1,2,4,23^
